# Nutritional and Inflammatory Markers and In-hospital Mortality in Geriatric Hip Fractures: A Retrospective Study

**DOI:** 10.5152/eurasianjmed.2026.261505

**Published:** 2026-10-26

**Authors:** Bahar Özaltaş, Arzu Yıldırım Ar, Süheyla Abitağaoğlu, Öznur Demiroluk, Barış Yılmaz

**Affiliations:** 1 Department of Anaesthesiology and Reanimation, Fatih Sultan Mehmet Training and Research Hospital, İstanbul, Türkiye; 2 Department of Anaesthesiology and Reanimation, Sultan Abdulhamid Han Health Application and Research Hospital, Health Sciences University, İstanbul, Türkiye; 3 Department of Orthopedics and Traumatology, Fatih Sultan Mehmet Training and Research Hospital, İstanbul, Türkiye

**Keywords:** Geriatric nutrition, hip fracture, inflammatory markers, in-hospital mortality, prognosis

## Abstract

**Background:**

Geriatric hip fractures are associated with high morbidity and mortality. Malnutrition and systemic inflammation are common in this population and may adversely affect outcomes. This study evaluated the associations of nutritional and inflammatory markers with in-hospital mortality.

**Methods:**

Geriatric patients admitted with hip fractures between January 2018 and January 2022 were retrospectively analyzed. Nutritional status was assessed using the Prognostic Nutritional Index, Nutritional Risk Screening (NRS-2002), and Geriatric Nutritional Risk Index. Inflammatory markers included the C-reactive protein/albumin ratio (CAR), neutrophil-to-lymphocyte ratio, and platelet-to-albumin ratio. The primary endpoint was all-cause in-hospital mortality during the index hospitalization.

**Results:**

: Among 400 patients, the in-hospital mortality rate was 5.0% (n = 20), and 34.0% (n = 136) required intensive care unit admission. Non-survivors were significantly older, had lower body mass index, and higher NRS-2002 scores (all *P* < .050). In multivariable logistic regression models adjusted for age and sex, higher NRS-2002 scores remained independently associated with mortality (OR: 3.26-3.40; 95% CI: 1.51-7.18; all *P* ≤ .003), whereas CAR and PNI were not. Receiver operating characteristic curves analysis demonstrated the highest discriminatory ability for NRS-2002 (area under the curve: 0.685), followed by CAR (0.631) and PNI (0.568).

**Conclusion:**

: Nutritional Risk Screening-2002 was the only marker consistently associated with in-hospital mortality. Structured nutritional screening may provide more clinically meaningful short-term risk stratification than isolated laboratory-derived indices in geriatric hip fracture patients.

**Main Points**

- Nutritional risk, particularly as assessed by Nutritional Risk Screening-2002, was the only marker consistently associated with in-hospital mortality in geriatric patients with hip fractures.
- Intensive care unit admission was strongly associated with mortality, highlighting the importance of overall clinical severity in this patient population.
- Commonly used laboratory-derived indices (Prognostic Nutritional Index, Geriatric Nutritional Risk Index, C-reactive protein/albumin ratio, neutrophil-to-lymphocyte ratio, and platelet-to-albumin ratio) did not independently predict mortality after adjustment, suggesting limited value in acute prognostic assessment.
- Nutritional deterioration accompanied advancing age but did not fully explain mortality risk.
- Structured nutritional screening tools may provide more clinically meaningful risk stratification than isolated laboratory-derived biomarkers in the acute hip fracture setting.

## Introduction

Hip fractures constitute an important health problem associated with high rates of morbidity and mortality in the older adult population.^1^ The rising trend of global life expectancy has been accompanied by an increase in the occurrence of hip fractures among the older population, especially because of the growing number of older adults admitted to hospitals after experiencing falls or other traumatic events.^2^^,^^3^ There is a strong association between patient age and mortality; in non-cardiac surgeries, mortality is expected to increase 1.35-fold per decade.^4^ Geriatric hip fracture patients experience mortality rates of roughly 7% in-hospital, increasing to 11% at 6 months, 30% annually, and over 40% after 3 years, despite advancements in diagnosis, treatment, and complication management.^5^

Hip fractures induce a complex systemic inflammatory response characterized by activation of innate immunity, release of pro-inflammatory cytokines, acute-phase protein production, and transient immune dysregulation.^6^ In older adults, age-related immunosenescence may further amplify this response, contributing to increased vulnerability to adverse outcomes, prolonged recovery, and mortality.^4^ Therefore, inflammatory markers may provide additional prognostic information beyond traditional clinical variables.

Malnutrition constitutes a significant risk factor for hip fractures, subsequent functional decline, and increased mortality.^2^^,^^5^ Studies using different nutritional assessment tools in different populations have found malnutrition to range from 4% to 39.4% in geriatric patients with hip fractures during hospitalization.^7^ In older adults, malnutrition is associated with impaired recovery, prolonged hospitalization, and increased mortality. Addressing nutritional deficiencies in this population is essential for enhancing overall well-being.^8^^,^^9^

Inflammatory and nutritional markers have demonstrated potential as prognostic indicators for geriatric patients. Established nutritional screening tools such as the Prognostic Nutritional Index (PNI), Nutritional Risk Screening (NRS-2002), and Geriatric Nutritional Risk Index (GNRI) provide an assessment of nutritional status and have been investigated as potential prognostic indicators in older adults.^10^^,^^11^ Concurrently, inflammatory markers, such as the C-reactive protein/albumin ratio (CAR), neutrophil-to-lymphocyte ratio (NLR), and platelet-to-albumin ratio (PAR), indicate the body’s response to injury and inflammation.^12^^,^^13^

Although several nutritional and inflammatory markers have been examined separately in older surgical populations, comparative evidence evaluating structured nutritional screening tools and laboratory-derived inflammatory indices within the same geriatric hip-fracture cohort remains limited. In particular, it is unclear whether admission-based laboratory indices add prognostic value beyond clinically oriented nutritional screening tools for short-term in-hospital mortality.

Recent studies have suggested that lower PNI values and higher CAR levels may be associated with worse outcomes in older surgical patients, including those with hip fractures.^14^^,^^15^ Similarly, NLR and PAR have been investigated as candidate inflammatory biomarkers reflecting immune dysregulation and adverse clinical outcomes in various surgical populations.^16^^,^^17^ However, their clinical utility in geriatric hip fracture patients remains insufficiently defined.

The present study focused on CAR, NLR, and PAR because these biomarkers are inexpensive, routinely available at hospital admission, and reflect distinct yet complementary dimensions of the inflammatory response. C-reactive protein/albumin ratio combines information on systemic inflammation and nutritional status, NLR reflects immune system dysregulation through the balance between innate and adaptive immunity, and PAR integrates inflammatory activation with protein reserve.^6^^,^^16^ Although alternative indices, including the Systemic Immune-Inflammation Index and the Systemic Inflammation Response Index, have also been investigated in various clinical settings, priority was given to biomarkers that are routinely available at hospital admission and can be readily integrated into everyday clinical practice, enabling a direct comparison with established nutritional screening tools in geriatric hip fracture patients.

This study aimed to evaluate the prognostic significance of admission-based nutritional and inflammatory markers in geriatric patients hospitalized with hip fractures. Specifically, the study sought to (i) compare these markers across different age groups, (ii) examine their associations with in-hospital mortality, and (iii) assess their independent prognostic value for short-term in-hospital outcomes.

## Material and Methods

### Research Design

This retrospective study analyzed data from geriatric patients, 65 years of age and older, admitted to the İstanbul Fatih Sultan Mehmet Training and Research Hospital with hip fractures during the period from January 2018 to January 2022. This study was approved by the Clinical Research Ethics Committee of İstanbul Fatih Sultan Mehmet Training and Research Hospital (Approval No. 24.03.2022/46; Date: March 24, 2022). Patient data were anonymized to ensure confidentiality. Due to the retrospective design of the study, informed consent was waived by the ethics committee. Institutional permission was obtained prior to data collection, and the study was performed in accordance with the principles of the Declaration of Helsinki. All consecutive patients aged ≥ 65 years who were admitted with hip fracture during the study period were screened for eligibility. Patients were excluded if they were younger than 65 years, had documented malabsorption disorders, multiple trauma, or incomplete index-hospitalization data. A patient flow diagram was prepared to summarize the screening, exclusion, and final inclusion process (Figure 1). Participants were categorized into 3 age groups: 65-74 years (young-old), 75-84 years (middle-old), and 85 years and above (oldest-old). Exclusion criteria were (i) age < 65 years, (ii) documented malabsorption disorders (e.g., celiac disease, short bowel syndrome, chronic pancreatic insufficiency with steatorrhea), (iii) multiple trauma, and (iv) missing or incomplete index-hospitalization data in the medical record precluding outcome ascertainment. Because the study was based on retrospective hospital records and excluded patients with incomplete index-hospitalization data, selection bias cannot be excluded.

Data collection encompassed demographic details, nutritional indices, inflammatory markers, and clinical outcomes. Nutritional status was assessed at admission through the application of the PNI, NRS-2002, and GNRI. Inflammatory markers, such as the CAR, NLR, and PAR, were recorded for evaluating the presence and intensity of inflammation. All nutritional and inflammatory markers (PNI, NRS-2002, GNRI, CAR, NLR, PAR) were calculated from laboratory and clinical data obtained at admission. Prognostic Nutritional Index was calculated as 10 × serum albumin (g/dL) + 0.005 × total lymphocyte count (/mm^3^). Geriatric Nutritional Risk Index was calculated as 1.489 × serum albumin (g/L) + 41.7 × [actual body weight/ideal body weight]. C-reactive protein/albumin ratio was calculated as C-reactive protein/albumin ratio, NLR as neutrophil count/lymphocyte count, and PAR as platelet count/albumin ratio.

The study collected data on a range of clinical parameters, including preoperative, perioperative, and postoperative transfusion requirements, length of hospitalization, use of bone cement during surgery, need for vasopressor and/or inotrope, intensive care requirements and duration, and in-hospital mortality outcomes. Clinical severity was assessed using routinely available hospitalization indicators, including intensive care unit (ICU) admission and ICU length of stay; however, these variables were not included in the final multivariable models because they may reflect post-admission clinical deterioration rather than baseline prognostic status.

The authors used artificial intelligence–assisted technologies (Grammarly and Microsoft Word Editor) during the manuscript preparation process to improve the readability, grammar, and overall language of the manuscript. These tools were used solely for language editing and did not contribute to the scientific content, study design, data analysis, interpretation of the results, or the formulation of clinical or scientific conclusions. The authors critically reviewed and approved all revisions and take full responsibility for the accuracy and integrity of the manuscript.

### Outcomes

The primary outcome was in-hospital mortality during the index hospitalization, defined as death from any cause occurring between hospital admission for the index hip-fracture episode and discharge from that same hospitalization.

### Statistical Analysis

Statistical analyses were performed to investigate the associations between nutritional and inflammatory markers and in-hospital mortality and to evaluate their prognostic value in geriatric hip fracture patients. Continuous variables are expressed as mean ± SD or median (interquartile range) according to distribution and categorical variables as n (%). Group comparisons were performed using Student’s *t*-test, Mann–Whitney *U*-test, Analysis of Variance, Kruskal–Wallis test, chi-square test, or Fisher’s exact test, as appropriate.

Given the limited number of in-hospital mortality events, multivariable logistic regression analyses were performed using parsimonious models to minimize the risk of overfitting. Age and sex were retained as core adjustment variables, and NRS-2002 was included as the primary nutritional screening parameter. C-reactive protein/albumin ratio and PNI were subsequently evaluated in separate and combined models to assess their incremental prognostic value. Intensive care unit hospitalization and total hospitalization time were not included in the final models, as these variables may reflect disease progression during hospitalization rather than baseline prognostic status. Odds ratios (ORs), accompanied by 95% CIs, were provided to convey the magnitude and statistical significance of the associations.

All analyses modeled in-hospital mortality (yes/no) during the index hospitalization. Receiver operating characteristic (ROC) curves were constructed for NRS-2002, CAR, and PNI to assess discrimination for in-hospital mortality. The area under the curve (AUC) with optimal cut-off values based on the Youden index was recorded. A 2-sided *P* < .05 was considered statistically significant. Statistical analyses were performed using NCSS (Number Cruncher Statistical System), Version 2007 (Kaysville, Utah, USA).

## Results

A total of 400 patients were included in the analysis (mean age: 82.0 ± 7.7 years), and 69.3% were female. Sex-based comparisons are summarized in Table 1. Male patients exhibited poorer nutritional status, with lower PNI and GNRI values, and a more pronounced inflammatory profile, with higher CAR and NLR values (all *P* < .05). Intensive care unit admission was more frequent among males (44.7% vs. 29.2%, *P* = .003), whereas in-hospital mortality rates were similar between sexes (4.9% vs. 5.1%, *P* > .999). Among comorbidities, congestive heart failure (CHF), arrhythmia, and chronic obstructive pulmonary disease were significantly more common in men (all *P* < .01).

Age-stratified analyses (Table 2) showed worsening nutritional status with advancing age. Prognostic Nutritional Index decreased from 43.3 in the young-old group to 40.9 in the oldest-old group (*P* = .018), whereas NRS-2002 increased from 2.0 to 2.4 (*P* = .001). Intensive care unit admission rates also increased across age groups (19.7% vs. 43.7%, *P* = .001). Although mortality rates increased numerically from 2.8% to 7.2%, this trend was not statistically significant (*P* = .123).

Comparisons according to in-hospital mortality status are presented in Table 3. Non-survivors were older (85.5 vs. 81.9 years, *P* = .037), had lower body mass index (24.1 vs. 26.8 kg/m^2^, *P* = .024), and higher NRS-2002 scores (2.75 vs. 2.25, *P* = .006). They also had longer hospital stays (22.0 vs. 11.1 days, *P* = .004), markedly higher ICU admission rates (90% vs. 31%, *P* < .001), and longer ICU stays (11.8 vs. 4.4 days, *P* = .021). Higher American Society of Anesthesiologists classification, CHF, atrial fibrillation, and arrhythmia were also more common among non-survivors (all *P* ≤ .005). In contrast, PNI, GNRI, CAR, NLR, and PAR did not differ significantly between groups.

Parsimonious multivariable logistic regression analyses are presented in Table 4. After adjustment for age and sex, higher NRS-2002 score remained consistently associated with in-hospital mortality across all models (OR: 3.26-3.40; 95% CI: 1.51-7.18; all *P* ≤ .003). The addition of CAR and PNI did not improve mortality prediction, as neither CAR (OR: 1.02-1.05, *P* ≥ .732) nor PNI (OR = 0.99, *P* ≥ .673) demonstrated independent associations with mortality.

Receiver operating characteristic curve analysis (Figure 2) demonstrated that NRS-2002 had the highest discriminatory performance for predicting in-hospital mortality (AUC = 0.685), followed by CAR (AUC = 0.631) and PNI (AUC = 0.568).

## Discussion

In this study, admission-based nutritional and inflammatory markers showed heterogeneous relationships with clinical outcomes in geriatric patients with hip fractures. Although nutritional status worsened with advancing age, NRS-2002 was the only marker that remained consistently associated with in-hospital mortality after adjustment. In contrast, PNI, GNRI, CAR, NLR, and PAR did not demonstrate independent associations with mortality. These findings suggest that structured nutritional screening may provide more clinically meaningful short-term prognostic information than isolated laboratory-derived biomarkers in this acute setting.

An important finding of the present study was the relatively stronger performance of NRS-2002 compared with the other evaluated indices. Nutritional Risk Screening-2002 integrates information related to both nutritional impairment and disease severity, which may be particularly relevant in geriatric hip fracture patients, where acute trauma, frailty, comorbidity burden, and perioperative stress interact. Although its discriminatory performance was modest, NRS-2002 consistently outperformed the laboratory-derived indices. This interpretation is biologically plausible, as previous studies have demonstrated that poor nutritional status is associated with adverse outcomes in older adults with hip fractures.^5^^,^^7^^,^^9^

The findings are also consistent with previous reports showing that nutritional risk is highly prevalent in older adults. Saka et al^18^ reported that 31% of older adults were at risk of malnutrition and 13% had established malnutrition. Similar findings have been documented in other populations.^19^^-^^22^ In the cohort, 28.5% of patients were classified as being at nutritional risk according to NRS-2002, supporting the importance of routine nutritional screening at hospital admission.

Prognostic Nutritional Index decreased across age groups, suggesting deterioration of nutritional reserve with advancing age. However, PNI was not independently associated with in-hospital mortality after adjustment. Previous studies have reported associations between lower PNI values and adverse outcomes in various populations.^23^ However, PNI primarily reflects albumin concentration and lymphocyte count, both of which may be influenced by acute inflammation, hydration status, trauma response, and perioperative stress. Therefore, PNI may reflect background nutritional vulnerability but may be less informative for predicting short-term mortality after acute hip fracture.

Previous studies have also reported an association between elevated CAR and mortality in older hip fracture patients.^24^ In contrast, CAR was not independently associated with in-hospital mortality in the adjusted models, and its discriminatory performance was limited. This discrepancy may be related to differences in outcome definitions, patient selection, perioperative management, and the use of a single admission-based measurement. Although CAR may capture acute inflammatory burden, it may not adequately reflect the multidimensional clinical severity that influences short-term mortality in geriatric hip fracture patients.

Similarly, GNRI, NLR, and PAR were not independently associated with in-hospital mortality in the cohort. Several explanations may account for these findings. First, GNRI primarily reflects chronic nutritional status, whereas short-term in-hospital outcomes may be more strongly influenced by acute illness severity and perioperative stress. Second, NLR and PAR are sensitive to the timing of measurement relative to trauma, surgery, intercurrent infection, and transfusion requirements. Therefore, a single admission-based assessment may not adequately capture the dynamic inflammatory response occurring after hip fracture. Residual confounding due to unavailable perioperative variables may have also influenced these findings.^9^

The strong unadjusted association between ICU admission and mortality observed in the cohort underscores the central role of overall clinical severity in this population. Similar observations have been reported previously.^6^ However, ICU admission and ICU length of stay were not included in the final regression models because these variables may reflect post-admission clinical deterioration rather than baseline prognostic status. This distinction is important when interpreting short-term mortality risk in retrospective hip fracture cohorts.

This study has several limitations. First, its retrospective single-center design limits generalizability and introduces the possibility of selection bias, particularly because patients with incomplete index-hospitalization records were excluded. Second, the number of in-hospital mortality events was relatively small. Therefore, parsimonious regression models were used and the number of covariates was limited; nevertheless, the possibility of overfitting, unstable estimates, and type II error cannot be fully excluded. In addition, important perioperative variables—including time to surgery, surgical procedure, anesthesia type, intraoperative instability, and postoperative complications—were unavailable and may have confounded the observed associations. Frailty status, cognitive impairment, and fracture subtype were also unavailable in the retrospective records and could not be evaluated. Finally, biomarker assessment was based solely on admission values without serial measurements. Additionally, serum vitamin B12, folate, thiamine, vitamin B6, and vitamin D levels were not routinely available in the retrospective records; therefore, their potential contribution to mortality risk could not be assessed.

Overall, these findings indicate that admission-based nutritional and inflammatory markers should not be interpreted uniformly in geriatric hip fracture patients. While age-related deterioration in nutritional status was evident, NRS-2002 appeared to have greater short-term prognostic relevance than PNI, GNRI, CAR, NLR, or PAR. Prospective multicenter studies incorporating frailty, cognitive status, perioperative variables, and serial biomarker measurements are needed to validate these findings.

Among the evaluated nutritional and inflammatory markers, NRS-2002 was the only marker consistently associated with in-hospital mortality in geriatric patients with hip fractures. Intensive care unit hospitalization was strongly associated with mortality and likely reflected overall clinical severity rather than an independent baseline prognostic factor. In contrast, PNI, GNRI, CAR, NLR, and PAR did not demonstrate independent associations with mortality after adjustment. These findings suggest that structured nutritional screening tools may provide more clinically meaningful prognostic information than isolated laboratory-derived biomarkers in the acute hip fracture setting. Prospective multicenter studies with larger sample sizes and more comprehensive perioperative data are warranted to validate these findings.

## Figures and Tables

**Figure 1.**
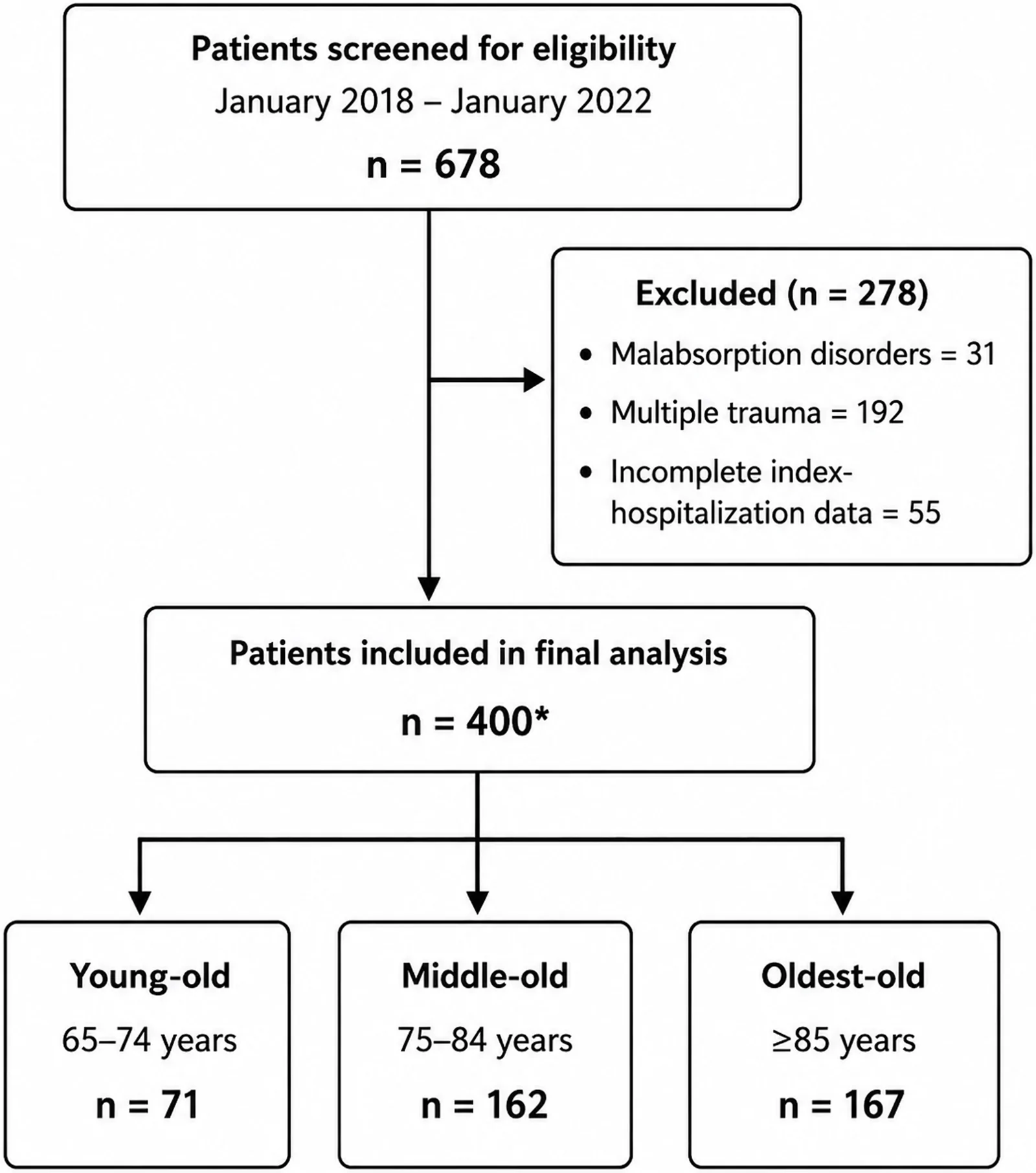
Flow diagram of patient selection and inclusion process. Flow diagram illustrating patient screening, exclusion, and final inclusion in the study. Among 678 patients screened between January 2018 and January 2022, 278 were excluded because of documented malabsorption disorders (n = 31), multiple trauma (n = 192), or incomplete index-hospitalization data (n = 55). The final cohort consisted of 400 geriatric patients categorized into young-old (65-74 years), middle-old (75-84 years), and oldest-old (≥85 years) groups.

**Figure 2.**
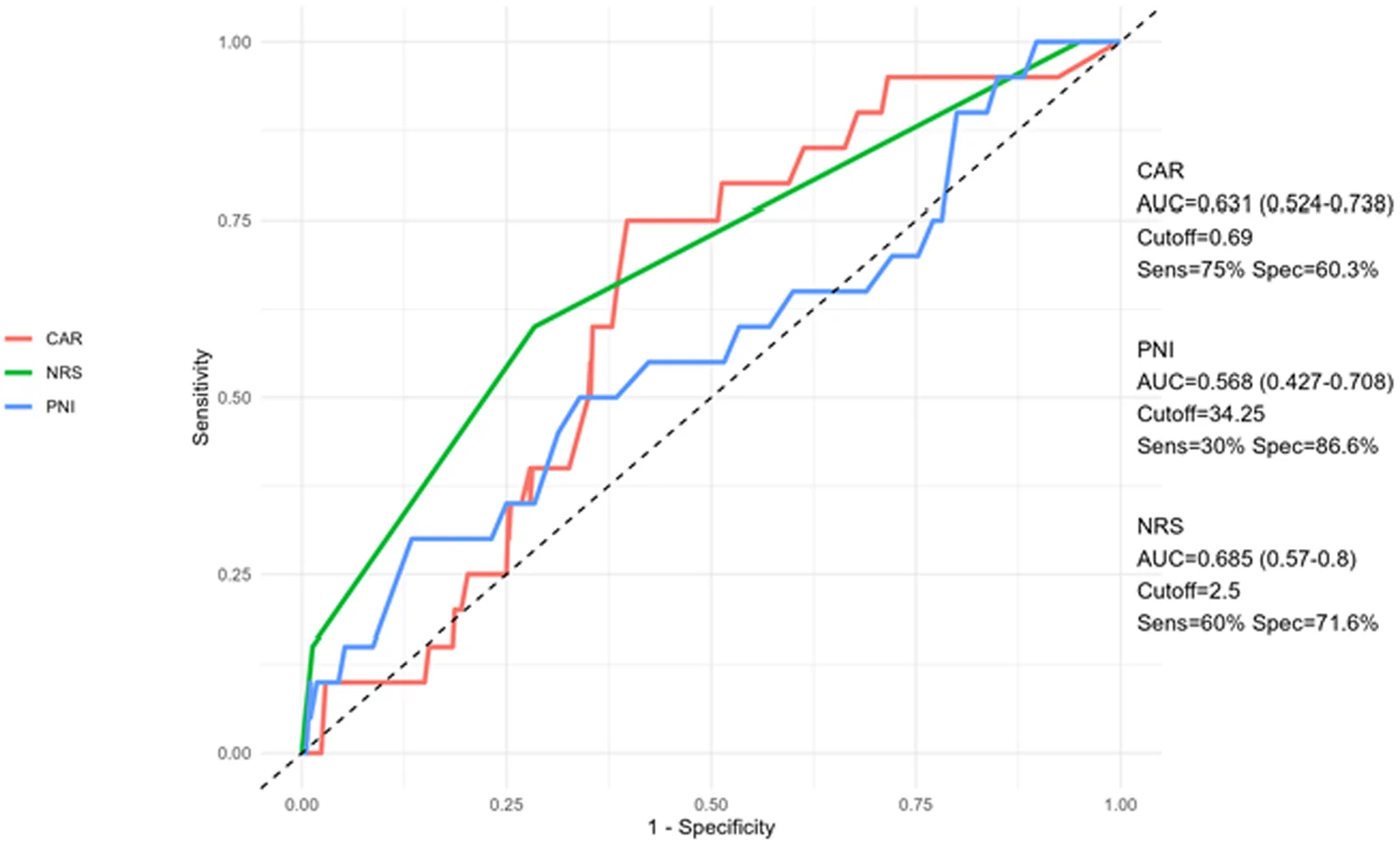
Receiver operating characteristic (ROC) curves for predicting in-hospital mortality using NRS-2002, CAR, and PNI. NRS-2002 demonstrated the highest discriminatory performance (AUC = 0.685), followed by CAR (AUC = 0.631) and PNI (AUC = 0.568). AUC, area under the curve; CAR, C-reactive protein-to-albumin ratio; NRS-2002, Nutritional Risk Screening-2002; PNI, Prognostic Nutritional Index.

**Table 1.** Patient Demographics and Baseline Characteristics

	Overall, n (%) = 400	Female, n (%) = 277	Male, n (%) = 123	*P*
Age (years)*	82.03 ± 7.74	82.29 ± 7.69	81.43 ± 7.84	.305
BMI (kg/m^2^)*	26.62 ± 5.25	27.99 ± 5.35	23.55 ± 3.42	**<.001**
PNI*	42.03 ± 8.17	42.64 ± 8.60	40.67 ± 6.95	**.026**
NRS-2002*	2.27 ± 0.58	2.25 ± 0.59	2.33 ± 0.55	.161
GNRI*	99.33 ± 14.59	102.11 ± 15.05	93.06 ± 11.25	**<.001**
CAR*	1.13 ± 1.51	1.02 ± 1.40	1.40 ± 1.72	**.021**
NLR*	7.03 ± 5.97	6.14 ± 4.72	9.04 ± 7.77	**<.001**
PAR*	6.88 ± 3.03	7.02 ± 3.08	6.57 ± 2.92	.173
Hb (g/dL)*	11.19 ± 1.87	10.96 ± 1.77	11.71 ± 2.00	**<.001**
Total hospitalization time (days)*	11.64 ± 10.74	11.42 ± 11.28	12.13 ± 9.43	.544
ICU hospitalization	136 (34)	81 (29.2)	55 (44.7)	**.003**
ICU hospitalization time (days)*	5.53 ± 10.7	5.91 ± 11.52	5.00 ± 9.48	.637
In-hospital mortality	20 (5.0)	14 (5.1)	6 (4.9)	>.999
Comorbidity				
ASA				**.006**
1	3 (0.8)	3 (1.1)	0 (0)	
2	21 (5.3)	20 (7.2)	1 (0.8)	
3	277 (69.3)	195 (70.4)	82 (66.7)	
4	99 (24.8)	59 (21.3)	40 (32.5)	
HT	312 (78)	223 (80.5)	89 (72.4)	.069
DM	105 (26.3)	75 (27.1)	30 (24.4)	.573
CAD	92 (23)	60 (21.7)	32 (26)	.912
CHF	75 (18.8)	39 (14.1)	36 (29.3)	**<.001**
AF	62 (15.5)	43 (15.5)	19 (15.4)	.984
Arrhythmia	149 (37.3)	91 (32.9)	58 (47.2)	**.006**
COPD	87 (21.8)	49 (17.7)	38 (30.9)	**.003**
Dementia	94 (23.5)	67 (24.2)	27 (22)	.626
Malignancy	32 (8)	19 (6.9)	13 (10.6)	.207

Bold values indicate statistically significant differences (*P* < .05)

AF, atrial fibrillation; ASA, American Society of Anesthesiologists classification; BMI, body mass index; CAD, coronary artery disease; CAR, C-reactive protein/albumin ratio; CHF, congestive heart failure; COPD, chronic obstructive pulmonary disease; DM, diabetes mellitus; GNRI, Geriatric Nutritional Risk Index; Hb, hemoglobin; HT, hypertension; ICU, intensive care unit; NLR, neutrophil-to-lymphocyte ratio; NRS-2002, Nutritional Risk Screening; PAR, platelet-to-albumin ratio; PNI, Prognostic Nutritional Index.

*Values are presented as mean ± SD for continuous variables and as n (%) for categorical variables. Statistically significant P-values (<.05) are highlighted.

**Table 2.** Age-Specific Comparison of Nutritional Indices, Inflammatory Markers, Perioperative Parameters, and Mortality Outcomes

	Age Groups			*P*
	Young-Old, n (%) = 71	Middle-Old, n (%) = 162	Oldest Old, n (%) = 167	
PNI*	43.26 ± 7.49	42.71 ± 9.07	40.86 ± 7.39	**.018**
NRS-2002*	1.99 ± 0.59	2.31 ± 0.53	2.36 ± 0.57	**.001**
GNRI*	100.21 ± 15.02	98.90 ± 14.52	99.37 ± 14.55	.818
CAR*	0.95 ± 1.34	1.10 ± 1.42	1.25 ± 1.67	.467
NLR*	6.64 ± 5.65	7.44 ± 7.12	6.80 ± 4.78	.448
PAR*	6.67 ± 2.51	6.89 ± 3.37	6.97 ± 2.91	.574
Hb (g/dL)*	11.81 ± 1.87	11.02 ± 1.92	11.10 ± 1.79	**.009**
Total hospitalization time (days)*	11.42 ± 13.20	11.45 ± 11.23	11.92 ± 9.02	.156
Hospitalization outcomes				.123
Discharged	69 (97.2)	150 (92.6)	146 (87.4)	
Referred	0 (0.0)	6 (3.7)	9 (5.4)	
In-hospital mortality	2 (2.8)	6 (3.7)	12 (7.2)	
ICU hospitalization	14 (19.7)	49 (30.2)	73 (43.7)	**.001**
ICU hospitalization time (days)*	5.64 ± 14.28	5.51 ± 12.09	5.53 ± 8.98	.169

*Values are expressed as mean ± SD for continuous variables and as n (%) for categorical variables. Age groups were defined as follows: young-old (65-74 years), middle-old (75-84 years), and oldest-old (≥85 years). Significant *P*-values (<.05) are highlighted.

CAR, C-reactive protein/albumin ratio; GNRI, Geriatric Nutritional Risk Index; Hb, hemoglobin; ICU, intensive care unit; NLR, neutrophil-to-lymphocyte ratio; NRS-2002, Nutritional Risk Screening; PAR, platelet-to-albumin ratio; PNI, Prognostic Nutritional Index.

**Table 3.** Comparison of Nutritional and Inflammatory Markers by In-hospital Mortality Status

Characteristic	Survivors, n (%) = 380	Non-survivors, n (%) = 20	*P*
Age (years)*	81.85 ± 7.74	85.45 ± 7.01	**.037**
BMI (kg/m^2^)*	26.76 ± 5.24	24.10 ± 4.76	**.024**
PNI*	42.15 ± 8.21	39.78 ± 7.25	.170
NRS-2002*	2.25 ± 0.56	2.75 ± 0.72	**.006**
GNRI*	99.62 ± 14.50	93.75 ± 15.65	.116
CAR*	1.12 ± 1.51	1.45 ± 1.56	.359
NLR*	6.88 ± 5.73	9.87 ± 9.28	.170
PAR*	6.89 ± 3.00	6.76 ± 3.70	.877
Hb (g/dL)*	11.2 ± 1.9	11.4 ± 2.0	.676
Total hospitalization time (days)*	11.09 ± 10.21	22.00 ± 15.02	.004
ICU hospitalization	118 (31)	18 (90)	**<.001**
ICU hospitalization time (days)*	4.41 ± 10.05	11.79 ± 12.23	**.021**
ASA			**<.001**
1	3 (0.8)	0 (0)	
2	21 (5.5)	0 (0)	
3	270 (71)	7 (35)	
4	86 (23)	13 (65)	
HT	295 (78)	17 (85)	.618
DM	98 (26)	7 (35)	.515
CAD	90 (24)	2 (10)	.252
CHF	66 (17)	9 (45)	**.005**
AF	54 (14)	8 (40)	**.005**
Arrhythmia	135 (36)	14 (70)	**.004**
COPD	81 (21)	6 (30)	.522
Dementia	87 (23)	7 (35)	.330
Malignancy	28 (7.4)	4 (20)	.108

AF, atrial fibrillation; ASA, American Society of Anesthesiologists classification; BMI, body mass index; CAD, coronary artery disease; CAR, C-reactive protein/albumin ratio; CHF, congestive heart failure; COPD, chronic obstructive pulmonary disease; DM, diabetes mellitus; GNRI, Geriatric Nutritional Risk Index; Hb, hemoglobin; HT, hypertension; ICU, intensive care unit; NLR, neutrophil-to-lymphocyte ratio; NRS-2002, Nutritional Risk Screening; PAR, platelet-to-albumin ratio; PNI, Prognostic Nutritional Index.

*Values are presented as mean ± SD for continuous variables and as n (%) for categorical variables. Statistically significant *P* values (<.05) are highlighted.

**Table 4.** Parsimonious Multivariable Logistic Regression Models for In-Hospital Mortality

Characteristic	Model 1			Model 2			Model 3			Model 4		
	OR	95% CI	*P*	OR	95% CI	*P*	OR	95% CI	*P*	OR	95% CI	*P*
Age	1.04	0.97- 1.11	.275	1.04	0.97- 1.11	.277	1.04	0.97- 1.11	.301	1.04	0.97-1.11	.299
Sex (male)	0.96	0.35-2.63	.937	0.95	0.35- 2.60	.918	0.94	0.34-2.58	.905	0.94	0.34-2.57	.899
NRS-2002 score	3.40	1.60-7.18	**.001**	3.31	1.55-7.10	**.002**	3.29	1.54-7.04	**.002**	**3.26**	**1.51-7.03**	**.003**
CAR				1.05	0.79-1.39	.760				1.02	0.76-1.39	.732
PNI							0.99	0.93-1.05	.673	0.99	0.93-1.05	.874

Data are presented as odds ratio (OR) with 95% CI.

Model 1 included age, sex, and NRS-2002 score. Model 2 additionally included CAR, model 3 additionally included PNI, and model 4 included both CAR and PNI. Models were kept parsimonious because of the limited number of in-hospital mortality events.

CAR, C-reactive protein-to-albumin ratio; NRS-2002, Nutritional Risk Screening-2002; PNI, Prognostic Nutritional Index.

## Data Availability

The data that support the findings of this study are available on request from the corresponding author.
